# Superoxide signalling and antioxidant processing in the plant nucleus

**DOI:** 10.1093/jxb/erae090

**Published:** 2024-03-09

**Authors:** Barbara Karpinska, Christine H Foyer

**Affiliations:** School of Biosciences, College of Life and Environmental Sciences, University of Birmingham, Edgbaston, Birmingham, UK; School of Biosciences, College of Life and Environmental Sciences, University of Birmingham, Edgbaston, Birmingham, UK; VIB-Ghent University, Belgium

**Keywords:** Ascorbate, chloroplasts, glutathione, meristems, mitochondrial Complex 1, nitric oxide, RBOH-type NADPH oxidases, superoxide dismutases

## Abstract

The superoxide anion radical (O_2_·^−^) is a one-electron reduction product of molecular oxygen. Compared with other forms of reactive oxygen species (ROS), superoxide has limited reactivity. Nevertheless, superoxide reacts with nitric oxide, ascorbate, and the iron moieties of [Fe–S] cluster-containing proteins. Superoxide has largely been neglected as a signalling molecule in the plant literature in favour of the most stable ROS form, hydrogen peroxide. However, superoxide can accumulate in plant cells, particularly in meristems, where superoxide dismutase activity and ascorbate accumulation are limited (or absent), or when superoxide is generated within the lipid environment of membranes. Moreover, oxidation of the nucleus in response to environmental stresses is a widespread phenomenon. Superoxide is generated in many intracellular compartments including mitochondria, chloroplasts, and on the apoplastic/cell wall face of the plasma membrane. However, nuclear superoxide production and functions remain poorly documented in plants. Accumulating evidence suggests that the nuclear pools of antioxidants such as glutathione are discrete and separate from the cytosolic pools, allowing compartment-specific signalling in the nucleus. We consider the potential mechanisms of superoxide generation and targets in the nucleus, together with the importance of antioxidant processing in regulating superoxide signalling.

## Teaser

Superoxide is emerging as a key player in stem cell regulation and plant development, as well as a pivotal signal in the nucleus. Like H_2_ O_2_ , superoxide is central to cell signalling.

## Introduction

Superoxide (O_2_·^−^), which is an anion radical, is produced by the one-electron reduction of molecular oxygen. In aqueous media, protonation of superoxide can result in the formation of the uncharged hydroperoxyl radical (HOO·; p*K*_a_ of 4.8). Hence, the anion radical form is by far the predominant species at physiological pH ranges. A second reduction of superoxide would require the energetically unfavourable compression of two full negative charges, and hence superoxide is generally considered to be a better reducing agent than an oxidant. Superoxide does not readily cross lipid membranes and has a long lifetime within lipid environments. The same may also be true of certain membrane-less organelles (Fuentes-Lemus and Davies, 2023).

Superoxide is relatively unreactive towards most cellular molecules. However, the superoxide-dependent reduction of free Fe^3+^ releases Fe^2+^ from ferritin, and superoxide interacts with positively charged iron porphyrins and enzymes that contain iron–sulfur clusters. Such interactions can lead to the inactivation of enzymes that are important in energy production and amino acid metabolism, such as the tricarboxylic acid cycle enzyme aconitase (Scandroglio *et al.*, 2014). The superoxide-dependent inactivation of aconitase leads to enhanced glycolysis relative to oxidative phosphorylation in cellular energy generation. Moreover, nuclear [Fe–S] cluster-containing proteins are direct targets of superoxide. For example, superoxide-dependent oxidation of the [Fe–S] cluster in Repressor of Silencing 1 (ROS1) regulates meristem function and fate (Wang *et al.*, 2023, Preprint).

Reactive nitrogen species (RNS), such as nitric oxide (·NO), nitrogen dioxide (·NO_2_), *S*‐nitrosothiols (SNOs), and peroxynitrite (ONOO^−^), often function together with reactive oxygen species (ROS) in plant responses to biotic and abiotic stresses. For example, NO and ROS play essential signalling roles in the orchestration of systemic acquired resistance (SAR) to pathogens (Wang *et al.*, 2014). The NO synthase (NOS) enzymes that are the primary producers of NO in animals (Zhang *et al.*, 2023) are absent from plants. Although NOS-like activity has been reported in plants (Astier *et al.*, 2018), nitrate reductase appears to be the main source of NO production, for example in plant immunity responses (Vitor *et al.*, 2013). Thus, although NO is able to regulate transcription factors and chromatin-modifying histone deacetylase functions, there is no known mechanism of NO production in the nucleus. NO-dependent regulation of epigenetic mechanisms and nuclear gene expression must therefore be achieved by NO import into the nucleus (Wurm and Lindermayr, 2021). When NO and superoxide are produced simultaneously, they react together at a diffusion-controlled rate to form peroxynitrite. Peroxynitrite is a primary agent responsible for post-translational protein modifications, particularly the nitrosylation of active site tyrosine residues in enzymes, as well as the nitrosylation of glutathione (GSH) and other cellular thiols. Peroxynitrite can cross cell membranes and thus enter the nucleus (Islam *et al.*, 2015). However, this highly reactive and powerful oxidant is unstable, decomposing to nitrate and dioxygen. We thus focus the following discussion on superoxide as a primary oxidant, whose abundance in different plant cell compartments is controlled by the expression of superoxide dismutase (SOD) and the abundance of low molecular weight antioxidants. In particular, we highlight recent evidence suggesting that O_2_·^−^ functions as a physiological, local signalling molecule in the nucleus.

## Superoxide as a signalling molecule

Superoxide, O_2_·^–^, is highly soluble in water, where it is strongly solvated by four tightly hydrogen-bonded water molecules. The protonated form of superoxide, hydroperoxyl, HO_2_, is a weak acid (p*K*_a_ of 4.8). Hence, the predominant species present in aqueous solutions at pH 7 is the small O_2_·^–^ anion together with its strongly associated four water molecules. The O_2_·^–^ anion is the substrate for the SOD enzymes.

Both O_2_·^–^ and HO_2_ are kinetically competent one-electron reductants. However, in most reactions only HO_2_ (and not O_2_·^–^) is the kinetically competent one-electron oxidant because of the need for either a proton or a coordinated metal ion to stabilize the peroxide dianion, O_2_^2–^, as it is formed (Sheng *et al.*, 2014). The disproportionation reaction is fastest at pH 4.8. In this situation, the concentrations of HO_2_ and O_2_·^–^ are equal, the former acting as an oxidant and the latter as a reductant. At high pH values, where the predominant species is O_2_·^–^, where two superoxide anions repel each other and the unshielded O_2_^2–^ is unstable, the disproportionation reaction cannot proceed and so O_2_·^–^ is quite stable. While superoxide has the thermodynamic capacity to be a strong oxidant, it is generally not reactive with most cellular components. However, labile iron–sulfur clusters in the reduced state are rapidly and irreversibly oxidized by reaction with superoxide. Although it is likely that other superoxide targets remain to be discovered, labile iron–sulfur-containing species are considered to be major superoxide targets. Thus, superoxide is a selective oxidant, relatively unreactive with most components of cells, but highly reactive with some essential cellular components, particularly labile iron–sulfur-containing species. The lifetime of O_2_·^–^ is considerably greater in aprotic media compared with aqueous solutions, and it thus has a greater stability when generated in lipid membranes. Moreover, although superoxide has a low membrane permeability, it can pass through anion channels (Andrés *et al.*, 2023).

In contrast to the rate of spontaneous non-enzymatic dismutation, which is relatively low at physiological pH values (2 × 10^5^ M^−1^ s^−1^), the SOD-catalysed dismutation reaction occurs at the almost diffusion-limited rate (∼2 × 10^9^ M^–1^ s^–1^) (Sheng *et al.*, 2014). The lifetime of superoxide in biological systems is thus determined by the rate of chemical dismutation and the presence of SOD and low molecular weight antioxidants, particularly ascorbate. The rate constant for the reaction between ascorbic acid and superoxide (at pH 7.4) is estimated as 5.4 × 10 M^–1^ s^–1^ using the xanthine–xanthine oxidase system (Som *et al.*, 1983). In contrast, the rate constant of the bovine erythrocyte Cu,Zn-SOD was calculated to be 1000 times higher (4 × 10^9^ M^–1^ s^–1^; Gray and Carmichael, 1992). Hence, the lifetime of superoxide as a signalling molecule can be considered to depend on the presence of SODs and ascorbate, which essentially police this molecule.

## Superoxide production and processing in plant cells

Oxygen delivery is essential for plant cell functions largely because of its role in aerobic respiration and ATP production through oxidative phosphorylation and the tricarboxylic acid (TCA) cycle in the mitochondria. As a non-electrolyte, molecular oxygen can cross the lipid bilayer of membranes by passive diffusion, permeating directly through the bilayer. Oxygen diffusion in cells and across membranes is of vital importance, as it allows the mitochondria and organelles, including the nucleus, to receive the oxygen that they need to survive and function.

The electron transport carriers of the mitochondrial electron transport chain, particularly the NADH dehydrogenase complex (Complex 1), are a major source of superoxide in plant cells, as are also the chloroplast electron transport chain and the RBOH (respiratory burst oxidase homolog)-type NADPH oxidases (NOXs) of the plasma membrane (Moller, 2001; Dietz *et al.*, 2016; Foyer and Hanke, 2022; Lee *et al*, 2023; Miller and Mittler, 2023). Superoxide is also produced by other reactions, for example in ureide and nucleic acid catabolism by the enzymes xanthine oxidoreductase (XOR) and urate oxidase, and in sulfite oxidation by sulfite oxidase (Sandalio *et al.*, 2021).

Superoxide is generated by the photosynthetic electron transport chain, largely at PSI by the processes termed the ‘Mehler reaction’ or pseudocyclic electron flow (Foyer and Hanke, 2022). The extent of superoxide accumulation in chloroplasts is variable. For example, Arabidopsis and tobacco leaves have double the amount of superoxide under short-day growth conditions compared with long-day conditions (Michelet and Krieger-Liszkay, 2012). The reasons why superoxide accumulation is allowed to vary in such circumstances are unknown. Superoxide can also be produced in peroxisomes through the activities of enzymes such as peroxisomal XOR, which generates uric acid with concomitant superoxide generation, as part of purine base degradation in the nucleotide degradation pathway. In contrast, superoxide generation in the plant nucleus, particularly in response to environmental stress, has not been explored in the literature.

Superoxide levels are kept low in plant cells by the effective compartmentalization of oxygen reduction reactions and by the expression of SODs that have high affinities for superoxide. The primary function of SODs is to protect anaerobic organisms from ROS accumulation by scavenging O_2_·^−^. Nevertheless, SODs generate another, less reactive ROS form, hydrogen peroxide (H_2_O_2_). The role of SODs is therefore to limit O_2_·^−^ accumulation and diffusion distances. The SOD enzymes in plants are classified according to the catalytic metal ions they contain, namely MnSODs, FeSODs, NiSODs, and Cu,ZnSODs. Each type of SOD is localized in different intracellular compartments or extracellularly in the apoplastic space. The presence of SOD facilitates the tightly regulated and spatially oriented redox signalling through both O_2_·^−^ and H_2_O_2_.

Current concepts consider that H_2_O_2_ is the predominant form of ROS used in redox signalling. However, increasing evidence suggests that superoxide also fulfils important signalling roles in plants and animals. For example, superoxide acts as a signal in young mutant animals to trigger changes of gene expression that prevent or attenuate the effects of subsequent ageing (Yang and Hekimi, 2010). Moreover, superoxide accumulation is important in the determination of stem cell and meristematic cell fate in plants and animals (Kim *et al.*, 2009; Soldner *et al.*, 2009; Tsukagoshi *et al.*, 2010; Zeng *et al.*, 2017). In plants, superoxide plays also an important role in xylem cell wall expansion, remodelling, lignification, and the induction of programmed cell death (PCD; Marzec-Schmidt *et al.*, 2020). Reduction of superoxide yields the non-radical ROS form, H_2_O_2_. In the presence of free transition metals, superoxide and H_2_O_2_ can give rise to the highly reactive hydroxyl radicals (Richards *et al.*, 2015), which are required for processes such as cell wall loosening and growth.

Apoplastic superoxide accumulation catalysed by enzymes such as amine oxidases plays an important role in cell wall loosening, allowing cell elongation during seed germination (Bailly, 2019). Superoxide generation accumulation by the plasma membrane-bound RBOH enzymes is triggered upon perception of chemical and/or physical cues (Yeung *et al.*, 2018). Such responses are relatively well characterized in relation to the plant immune system and the orchestration of local and systemic defence processes. Plants employ cell surface-resident pattern recognition receptors (PRRs) and intracellular nucleotide-binding domain leucine-rich repeat (NLR) receptors to detect the presence of pathogen-derived pathogen-associated molecular patterns (PAMPs) and effectors, activating PAMP-triggered immunity (PTI) and effector-triggered immunity (ETI), respectively (Jones and Dangl, 2006). PTI and ETI can be triggered by a diverse range of PAMPs and effectors, respectively, each with a specific receptor (Barragan and Weigel, 2021). In addition, the release of host-derived damage-associated molecular patterns (DAMPs) that are sensed by various PRRs induces DAMP-triggered immunity (DTI; Tanaka and Heil, 2021). Some virulent pathogens use effectors or virulence factors to suppress PTI-associated ROS production. Microorganisms secrete effector proteins that can trigger a multitude of host defences depending on their target destination. Some effectors can pass through the nuclear envelope to reach targets in the nucleus, where they modify target gene expression either by subverting epigenetic modifications or by direct interference with the transcriptional machinery (Harris *et al.*, 2023). Several nuclear effectors modify phytohormone defence signalling and suppress immunity-associated host responses (Harris *et al.*, 2023). For example, the CRN63 and CRN115 effector proteins produced by *Phytophthora sojae* target a cytoplasm-localized catalase and re-localize with it to the host nucleus, thereby suppressing host cell death responses (Zhang *et al.*, 2015). Similarly, the HaRxL106 effector proteins from *Hyaloperonospora arabidopsidis* suppress host immunity by binding to the RADICAL-INDUCED CELL DEATH1 (RCD1) transcription factor and suppress the salicylic acid (SA)-induced transcriptional activation of defence genes (Wirthmueller *et al.*, 2018). Such studies illustrate the concept that ROS production and processing are key targets for manipulation by microorganisms. It remains to be demonstrated whether symbiotic and pathogenic microorganisms are able to directly target superoxide signalling in the nucleus.

Of the Arabidopsis hypoxia-inducible *RBOH* genes in Arabidopsis, *RBOHD* and *RBOHF* are considered to be the most important in many stress responses (Chen *et al.*, 2015; Liu *et al.*, 2017; Yeung *et al.*, 2018). Moreover, anoxia-driven ethylene accumulation drives Ca^2+^ and calcium-dependent protein kinase (CDPK)5/13-mediated phosphorylation and activation of RBOH in the roots. The accumulation of superoxide but not hydrogen peroxide in this situation was found to be important in providing spatial cues for development of aerenchyma (Dunand *et al.*, 2007; Wany and Gupta, 2018).

Oxygen is a diffusible signal that controls many processes in plant and animal development, particularly the activity of stem cells. Mammalian stem cells reside in a hypoxic microenvironment that is considered to limit oxidative stress (Li *et al.*, 2021). However, ROS participate in activation of stem cells, stimulating them to enter the cell cycle upon exit from quiescence (Lyublinskaya *et al.*, 2015). A similar situation occurs in plant cells, such as those in the root apical meristem (RAM; de Simone *et al.*, 2017). The RBOH proteins play a pivotal role in this regulation, but the pathways involved remain poorly understood (Chapman *et al.*, 2019). Knocking out RBOH and mitochondrial Complex 1 subunits caused very similar phenotypes in stem cell maintenance (Zeng *et al.*, 2017), suggesting that both RBOH and NADH‐dehydrogenase‐derived superoxide molecules are important in the control of stem cell fate maintenance.

## Oxidant and antioxidant functions in the plant cell nucleus

The nucleus is an organelle with a range of metabolic pathways and processes including DNA replication, transcription, and gene regulation. Accumulating evidence suggests that these metabolic pathways include enzymes that generate superoxide. Nuclear superoxide-producing enzymes are well characterized in human and animal cells. Of the enzyme families that use molecular oxygen, the 2-oxoglutarate-dependent dioxygenases are particularly important as they are involved in histone and DNA demethylation and hydroxylation reactions (Huang *et al.*, 2023). Interestingly, ascorbate is a specific cofactor for the Fe(II)- and 2-oxoglutarate-dependent dioxygenases that catalyse the addition of a hydroxyl group to various substrates (Wei *et al*., 2021). Ascorbate is also a cofactor for the ten–eleven translocation (TET1−3) family of Fe(II)-dependent dioxygenases in mammalian cells, which are responsible for the removal of cytosine methylation in DNA (Zhithovich, 2020). The TET enzymes are Fe(II)-dependent dioxygenases that catalyse a series of consecutive oxidations of 5-methylcytosine. No TET-like enzymes have as yet been identified in plants, although 5-methylcytosine oxidation products, particularly 5-hydroxymethylcytosine (5hmC), have been reported (Mahmood and Dunwell, 2019). In addition, NOX4 generates superoxide specifically in the nucleus of specific human cell types including human vascular endothelial cells (Kuroda *et al.*, 2005). It is possible that NOX isoforms are also trafficked to the plant nucleus, not least because an increasing number of proteins display the ability to re-localize to the cell nucleus, for example by vesicle transport. Some transcription factors such as ERF74 translocate from the plasma membrane to the nucleus in response to hypoxia to regulate the transcription of *RBOHD* (Kosmacz *et al.*, 2015). All Arabidopsis RBOH proteins are generally targeted to the plasma membrane; RBOHD also had a potential nuclear localization signal (Table 1).

**Table 1.** Predicted nuclear localization of RBOH proteins in *Arabidopsis thaliana*

NADPH oxidase	Locus ID	TAIR database (GO cellular component)	LOCALIZER nuclei localization prediction	NLSP nuclear localization signal prediction	Probability (0–1)
RBOHA	At5g07390	PL, MT, NU	Yes	120–136	0.8
RBOHB	At1g09090	PL	–	–	–
RBOHC	At5g51060	PL, NU	Yes	51–60	0.966
RBOHD	At5g47910	PL, GL, NU	Yes	42–50	0.737
RBOHG	At4g25090	PL, VC	–	–	–
RBOHE	At1g19230	PL, NU	Yes	–	–
RBOHF	At1g64060	PL	–	–	–
RBOHI	At4g11230	PL	Yes	–	–
RBOHH	At5g60010	PL, NU	–	–	–
RBOHJ	At3g45810	PL, NU	Yes	–	–

Predictions were performed using LOCALIZER 1.0.4 and NLSP (Nuclear Localization Signal Prediction) tool. The LOCALIZER program is based on the collection of plant NLSP signals (https://localizer.csiro.au) and employs a Hidden Markov model.

**Fig. 1.**
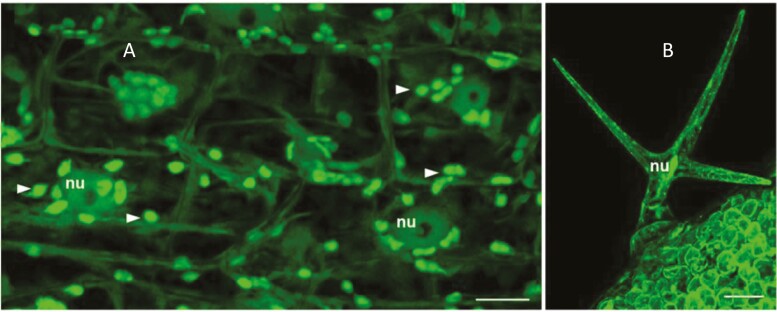
Localization of iron superoxide dismutase 1 (FSD1) in the nucleus. Localization of FSD1–green fluorescent protein (GFP) fusions in the chloroplasts and the nucleus (arrows) of mesophyll tissue (A) and trichomes (B) see Dvořák *et al*. (2021). Nu, nucleus; chloroplasts are indicated by arrows.

The animal and human cell SOD1 can rapidly re-localize to the nucleus in response to oxidative stress, where it acts as a nuclear transcription factor (Tsang *et al.*, 2014). SOD1 is localized in the nucleus under both normal and pathological conditions, where it functions as a regulatory protein in cell signalling, transcription, and ribosome biogenesis, contributing to oxidative stress responses and the control of growth (Xu *et al.*, 2022). The iron SOD (FSD1) is localized in the chloroplast stroma, the cytosol, and in nuclei (Fig. 1; Dvořák *et al.*, 2021; Melicher *et al.*, 2022). FSD1 was re-localized to the plasma membrane in response to salt stress (Dvořák *et al.*, 2021). A number of antioxidant enzymes are either localized in the nucleus, or can re-localize there in response to appropriate triggers (Foyer *et al*., 2020).

The majority of the macromolecular traffic, including proteins, in and out of the nucleus, occurs through the nuclear pores. This transport is mediated by the Karyopherin-β (or Kap) family of nuclear transport receptors (Wing *et al.*, 2022). Nucleocytoplasmic traffic is mediated by 20 different Kaps in mammalian systems, that function either across the nuclear pore complex into the nucleus (importins), out of the nucleus (exportins), or in both directions (biportins). The presence of SOD in this compartment strongly suggests that superoxide is produced within the plant cell nucleus. Moreover, the presence of SODs in the nuclei of animal and plant cells demonstrates that H_2_O_2_ is generated directly in the nuclei.

The generation of both superoxide and hydrogen peroxide in the nucleus facilitates compartment-specific control of redox regulation and signalling. The plant cell nucleus is rich in glutaredoxins (GRXs), thioredoxins (TRXs), thiol reductases, as well as other redox-regulated proteins that exert redox control over nuclear processes and functions, such as gene expression, chromatin remodelling, and epigenetics (He *et al.*, 2018; Martins *et al.*, 2018). It was recently suggested that the redox modulation of transcription factors by the co-regulatory CC-type GRXs that are called ROXY, and other CC-type plant-specific GRXs are important in the evolution of plant developmental controls (Zachgo, 2023). The ancestral role for CC-type GRXs in modulating the activities of TGACG-binding (TGA) transcription factors is well established in the literature. For example, ROXY1 and the TGA transcription factor called PERIANTHIA (PAN) govern root meristem activities, acting together in the control of root development (Maß *et al.* 2020). Moreover, ROXY1 co-localizes with the active form of RNA polymerase II in the nucleus (Gutsche *et al.*, 2017).

Ascorbate peroxidases (APXs) and catalase have been localized in plant cell nuclei, along with other antioxidant enzymes (Al-Hajaya *et al.*, 2022; Table 2). Moreover, high concentrations of ascorbate (16.3 mM) and GSH (6.4 mM) were reported in the nuclei of *Arabidopsis thaliana* leaves (Zechmann, 2011, 2014, 2020; Zechmann *et al.*, 2011, Liu *et al.*, 2019; Chen *et al.*, 2021).

**Table 2.** Enzymes that process reactive oxygen species (ROS) in the nucleus

Constructs	Method	Localization	Reference
35S:FDS1–GFP, 35S:GFP–FDS1	*Arabidopsis thaliana*—stable transgenic lines	Nucleus, chloroplast	Dvořák *et al*. (2021)
UBQ:CAT2–sfGFP, UBQ:sfGFP–CAT2	*Arabidopsis thaliana*, mesophyll protoplasts transfection	Nucleus, peroxisome	Al-Hajaya *et al*. (2022)
35S:APX1–GFP	Arabidopsis thaliana, mesophyll protoplasts transfection	Nucleus, plasma membrane	Liu *et al.* (2019)
35S:APX1–GFP	*Nicotiana benthamiana*, leaf infiltration	Nucleus, cytoplasm	Chen *et al.* (2021)
35S:GPX8–GFP	Onion epidermal cells bombardment	Nucleus, cytoplasm	Gaber *et al.* (2012)

Compartmentation of catalase 2 (CAT2), ascorbate peroxidase 1 (APX1), iron superoxide dismutase 1 (FSD1), and glutathione peroxidase 8 (GPX8) in the nucleus, detected using green fluorescence protein (GFP) fusions.

The concept that there is free diffusion of small molecules between the nucleus and cytosol through the nuclear pores is embedded in the literature. However, accumulating evidence suggests that the GSH pools of the nucleus are discrete. For example, the nuclear and cytosolic GSH pools are regulated independently during cell proliferation (Emmert *et al.*, 2023).

GSH co-localizes with nuclear DNA during the early stages of cells proliferation in plants and animals (Diaz Vivancos *et al.*, 2010). The recruitment and sequestration of GSH in the nucleus during the G_1_ and S phases of the cell cycle had a profound impact on gene expression. GSH was suggested to act as a ‘redox sensor’ at the onset of DNA synthesis, maintaining the nuclear architecture by providing the appropriate redox environment for DNA replication, and safeguarding DNA integrity, epigenetic controls, and protein degradation by nuclear proteasome (García-Giménez *et al.*, 2013). Live-cell GSH imaging revealed that the GSH concentration of the nucleus is highest during S phase, and steadily decreases until mitosis (Emmert *et al.*, 2023). Such findings demonstrate that the nuclear GSH pool is regulated independently of that elsewhere in the cell. Moreover, the existence of different GSH concentrations in the nucleus and the cytosol strongly suggests that GSH does not diffuse freely across the nuclear envelope or through nuclear pores (Emmert *et al.*, 2023). The same may be true for ascorbate, with the ascorbate pools in the nucleus and the cytosol regulated independently. Infection of Arabidopsis plants with *Pseudomonas syringae* increased the ascorbate contents of the nuclei followed by a strong decrease that was accompanied by accumulation of ROS (Großkinsky *et al.*, 2012). Similarly, a large decrease in the ascorbate (58%) content of the nucleus was observed within 12 h after the infection of Arabidopsis wild-type plants with *Botrytis cinerea* (Simon *et al.*, 2013). The nuclear GSH pool increased by 300%, together with ROS accumulation in the nucleus (Simon *et al.*, 2013). Exposure to abiotic stress conditions also causes a decrease in the nuclear ascorbate contents relative to the cytosol (Koffler *et al.*, 2014a, b, 2015). In addition, nuclear ascorbate levels were either increased or remained unchanged following exposure to abiotic stress situations in the ascorbate-deficient *vtc2-1* mutants, whereas the cytosolic ascorbate contents decreased in the wild type (Zechmann, 2018).

The presence of a redox cycle within the plant cell cycle, with controlled oxidation at the early stages, suggests that the redox state of the nuclei is precisely controlled to facilitate cell cycle progression (de Simone *et al.*, 2017). Such regulation would suggest that superoxide is generated within the plant cell nuclei in response to the factors that trigger cell cycle progression. Exposure to abiotic stresses such as heat shock causes rapid oxidation of the nucleus (Babbar *et al.*, 2021). Similarly, inhibitors that are commonly used to modulate chloroplast and mitochondrial electron transport pathways in order to study organelle to nucleus signalling all increase oxidation in the nuclei as well as the cytosol (Karpinska *et al.*, 2017). Moreover, the chloroplast inhibitor lincomycin and the mitochondrial inhibitor antimycin caused a greater oxidation in the nuclei of stomatal guard cells than the cytosol (Karpinska *et al.*, 2017). Stress-induced oxidation of nuclei is crucially important for the regulation of gene expression and as associated signal transduction pathways, as well as the control of nuclear thiol–disulfide redox states by nucleoredoxins, GRXs, and TRX1 (Kneeshaw *et al.*, 2017). For example, the pathogen-inducible oxidoreductase Nucleoredoxin 1 (NRX1) targets H_2_O_2_-scavenging enzymes, including catalases. NRX1 forms a mixed disulfide intermediate with catalase *in vivo*, protecting H_2_O_2_-scavenging activity (Kneeshaw *et al.*, 2017).

## Superoxide and cell to cell signalling

The ROS wave concept of cell to cell signalling that plays a pivotal role in local and systemic responses that underpin acclimation to stress (Waszczak *et al.*, 2018; Fichman *et al.*, 2022; Mittler *et al.*, 2022) requires an ‘activated ROS production’ state that is driven by RBOHs. The wave is propagated from cell to cell over long distances in order to activate gene expression that enhances the overall resilience of the plant to stress (Mittler *et al.*, 2022). The activation of RBOHs such as RBOHD and RBOHF was required to produce superoxide that is the essential driver of this process (Fichman *et al.*, 2022, 2023). The hydrogen peroxide receptor HPCA1 is required for the propagation of cell to cell ROS and calcium signals that underpin systemic signalling in response to different biotic and abiotic stresses (Fichman *et al.*, 2022). Thus, apoplastic H_2_O_2_ sensing and signalling are intrinsic to the systemic propagation of cell to cell ROS and calcium signals. However, such findings do not rule out a possible role for superoxide signalling and sensing in this process. Such pathways appear to have evolved as part of the quorum-sensing network in unicellular organisms. Evidence for the operation of the ROS wave pathway has now been presented in many organisms including unicellular algae and mammalian cells (Szechyńska-Hebda *et al.*, 2023; Fichman *et al.*, 2023).

## Organelle to nucleus signalling pathways

Retrograde signalling pathways link the functional state of mitochondria and chloroplasts to the nuclear gene expression in order to facilitate acclimation (Wang *et al.*, 2020). Organellar retrograde signals are initiated by ROS including superoxide and hydrogen peroxide, and further mediated by oxidized compounds and proteins. Mitochondrial signals initiate cellular responses to hypoxia, including the regulation of heat-shock proteins and other molecular chaperones, transporters, the ALTERNATIVE OXIDASE1a (AOX1a), and other components of the alternative respiratory chain, such as NAD(P)H DEHYDROGENASE B2 (NDB2; Wagner *et al.*, 2018). The mitochondrial signalling cascade that triggers these responses is called the ‘mitochondrial dysfunction stimulon’ (MDS; de Clercq *et al.*, 2013). The promotors of MDS genes are targeted by NAC (NO APICAL MERISTEM/ARABIDOPSIS TRANSCRIPTION ACTIVATION FACTOR/CUP-SHAPED COTYLEDON) transcription factors (de Clercq *et al.*, 2013), particularly ANAC013 and ANAC017/REGULATORS OF ALTERNATIVE OXIDASE 1a (RAO2), WRKY-type transcription factors (WRKY15/WRKY40/WRKY63; Vanderauwera *et al.*, 2012), and ABI4 (ABSISIC ACID INSENSITIVE 4; Giraud *et al.*, 2009). These are downstream regulators of mitochondrial retrograde signalling pathways. CYCLIN-DEPENDENT KINASE E1 (CDKE1/RAO1), which is part of the mediator complex that bridges DNA-bound transcription factors to RNA polymerase II, links signalling input to transcription, in order to regulate AOX1a expression as part of the retrograde signalling hub from mitochondria as well as chloroplasts (Blanco *et al.*, 2014). Significant overlap between chloroplast and mitochondrial retrograde signalling pathways has been observed involving common or shared components (Wang *et al.*, 2023, Preprint). ROS are a key component of the intracellular signalling pathways that regulate the expression of nuclear genes in an ANAC017-dependent manner (Huang *et al.*, 2016; Jurdak *et al.*, 2021). Two key pathways that operate at the interface between chloroplast to nucleus and mitochondria to nucleus signalling have been characterized, namely the SAL1–PAP signalling pathway and the RADICAL-INDUCED CELL DEATH 1 (RCD1)-dependent pathway (Shapiguzov *et al.*, 2019). These organelle to nucleus retrograde signalling pathways overlap or converge in regulating nuclear gene expression. The SAL1 phosphatase, which is localized in chloroplasts and mitochondria, degrades 3'-phosphoadenosine-5'-phosphosulfate (PAP) to AMP and inorganic phosphate. In the absence of SAL1, PAP accumulates and inhibits the activity of the cytosolic 5'–3' exoribonuclease XRN4 and the nuclear 5'–3' exoribonucleases XRN2 and XRN3. The XRNs play significant roles related to non-coding RNAs, including small RNA metabolism, which results in the modulation of nuclear gene expression (Ratti *et al.*, 2020).

RCD1 is localized in the cytosol and nucleus. The abundance, nuclear localization, thiol redox state, and oligomerization are regulated by chloroplast ROS accumulation in order to coordinate plant stress responses, phytohormone signalling pathways, and growth and development (Tao *et al.*, 2023). RCD1 integrates retrograde signalling from the chloroplasts and mitochondria through ANAC013 and ANAC017. The *rcd1* mutation compromised responses to chloroplast ROS accumulation and changed AOX expression, as well as mitochondrial respiration and energy metabolism. The genes misregulated in the *rcd1* mutant had a significant overlap with the genes affected by the PAP signalling pathway and the MDS genes, including those for AOX1a and the sulfotransferase SOT12, an enzyme that generates PAP (Shapiguzov *et al.*, 2019).

Mutations in the gene encoding the rice chloroplast-localized pseudouridine synthase (OSPUS 1-1) result in albino seedlings under low temperatures because of aberrant chloroplast ribosome biogenesis (Wang *et al.*, 2022). Overexpression of mitochondrial MnSOD rescues the phenotype, as does the suppressor protein of *ospus 1-1*, which encodes a mitochondrial pentapeptide repeat (PPR) protein, suggesting that superoxide is involved in the co-ordination of mitochondria to nucleus and chloroplast to nucleus signalling pathways.

## The role of superoxide in the regulation of plant development and stress responses

Superoxide generation is a pivotal driver of the cell cycle, pollen viability, microspore reprogramming towards sporophytic development, the regulation of female gametophyte patterning, and the maintenance of embryo sac polarity, as well as the prevention of self-pollination (de Simone *et al.*, 2017; Zur *et al.*, 2021; Sankaranarayanan *et al.*, 2020). ROS signals activate anaerobic core genes via the ERF-VII transcription factors (Sasidharan *et al.*, 2021). The RELATED TO AP-2.12 (RAP2.12) transcription factor regulates the expression of *HYPOXIA-RESPONSIVE UNIVERSAL STRESS PROTEIN 1* (*HRU1*) as well as *RBOHD* under hypoxia (Gonzali *et al.*, 2015). Oxygen levels can fall below 5% in the shoot apices and in the lateral root primordia (LRPs) of plants grown in air, leading to the expression of hypoxia-dependent genes (Shukla *et al.*, 2019). In contrast, hypoxia-responsive genes are not induced in the RAM under similar conditions (Eysholdt-Derzsó *et al.*, 2017; Shukla *et al.*, 2019). Oxygen gradients probably occur in the RAM, particularly because of the quiescent centre (QC) cells, which are deficient in antioxidants such as ascorbate and GSH (Jiang *et al.*, 2010). Flooding-induced hypoxic stress alters auxin flow and distribution in roots in a manner that can shift the redox state of the QC towards a more reduced environment, leading to QC activation and degradation of the meristem (Mira *et al.*, 2020).

The distinct spatiotemporal distribution of superoxide and hydrogen peroxide in shoot and root meristems is crucial for meristematic activities. Superoxide accumulates in meristems, where it serves to maintain cell divisions. In contrast, H_2_O_2_ is abundant in peripheral zones, promoting cell differentiation (Tsukagoshi *et al.* 2010; Zeng *et al.* 2017). Similarly, the spatial distribution of oxygen in plant organs is an important regulator of development, whose signalling functions connect developmental processes with metabolic activity (Weits *et al.*, 2023). Hypoxia is an established condition in the shoot apical meristem (SAM) that promotes leaf organogenesis which both increases and limits mitochondrial ROS production and also increases the activation of mitochondrial systems that remove ROS (Mailloux, 2020; Pucciariello and Perata, 2021). The oxygen signal is translated into transcriptional regulation through the N-degron pathway and the regulated expression of ERF-VII (ETHYLENE RESPONSE FACTOR—group VII) transcription factors that link metabolic controls to the regulation of development in plants. Mitochondrial oxygen consumption is also linked to oxygen sensing through the inner mitochondrial membrane UNCOUPLING PROTEIN 1 (UCP1) (Barreto *et al.*, 2022). This regulates nuclear gene expression by inhibiting the cytoplasmic PLANT CYSTEINE OXIDASE (PCO) branch of the PROTEOLYSIS 6 (PRT6) N-degron pathway linking mitochondrial and nuclear functions during plant development. Moreover, the CC-type GRXs are particularly important in the control of plant developmental processes, in organs and tissues where low oxygen signalling contributes to meristem functions (Zachgo, 2023). Redox post-translational modifications of TGA transcription factors, which are targets of ROXYs, regulate their functions in plant development. For example, ROXY1 interacts with the TGA transcription factor PAN in the nucleus to regulate petal formation in *A. thaliana* (Li *et al.*, 2009). ROXY1 proteins also co-localize with all three different RNA polymerase II (RNAPII) isoforms in the nucleus and so regulate RNAPII-mediated transcription (Maß *et al.* 2020).

Mitochondrial metabolism resumes during seed imbibition in order to provide energy for the germinating embryo (Paszkiewicz *et al.*, 2017). Ethylene-generated mitochondrial superoxide production and ROS accumulation regulate seed dormancy alleviation, via a mechanism that involves MRR, nuclear ROS accumulation, the expression of *AOX1a* and *ANAC013*, and the activation of the ethylene canonical pathway (Jurdak *et al.*, 2021).

The concept that ROS signals regulate plant development and growth is well established in the literature (Tsukagoshi *et al*, 2010; Considine and Foyer, 2021; Schmidt and Schippers, 2015; Schippers *et al.*, 2016). Distinct spatial patterns of superoxide and H_2_O_2_ accumulation have been reported in the root (Zhou *et al.*, 2020). Similarly, stem cells maintain high levels of superoxide and low H_2_O_2_, while differentiating cells show opposite patterns. Superoxide accumulation is maintained by the regulation of RBOH activities and the expression of mitochondrial AOX, together with inhibition of SOD expression (Zeng *et al.*, 2017). AOX functions as a pre-oxidant defence system, limiting superoxide production, when electron flow via the cytochrome electron transport chain is restricted. Superoxide was shown to decrease the levels of H_2_O_2_ in the stem cell by activating the expression of peroxidases (Zeng *et al.*, 2017).

Superoxide accumulates in the SAM and RAM, preserving meristematic activity (Tsukagoshi *et al.*, 2010) and stem cell fate (Zeng *et al.*, 2017). Prevention of superoxide accumulation causes the termination of stem cell fate. Similarly, superoxide accumulation in the RAM defines the identity of undifferentiated meristematic cells (Tsukagoshi *et al.*, 2010). The preservation of superoxide signals in Arabidopsis is facilitated by repressed expression of the seven SODs in the central zone (Zeng *et al.*, 2017). Similarly, treatment with c-Myc and Oct44 represses the three human and mice SODs and prevents the induced pluripotent stem cell stage in mammalian cells (Kim *et al.*, 2009; Soldner *et al.*, 2009). The UPBEAT1 (UPB1) transcription factor mediates the balance between superoxide and H_2_O_2_ in the root in a manner that regulates the transition from cell proliferation to cell expansion and differentiation (Tsukagoshi *et al*, 2010). UPB1 regulates the H_2_O_2_ content in the root apex by inhibiting the expression of class III peroxidases which are able to produce superoxide in the elongation zone which establishes the ROS gradient distribution in the root meristem. The mutation of an ATP-dependent mitochondrial protease, AtFTSH4, causes oxidation in the cells in the SAM at 30 °C and affects the morphology of the mitochondria and functions of the SAM (Dolzblasz *et al.*, 2016).

Superoxide and hydrogen peroxide appear to fulfil antagonistic roles in plant stem cell regulation, which were established by distinct spatiotemporal patterns of ROS-metabolizing enzymes, particularly in roots (Dunard *et al.*, 2007; Eljebbawi *et al.*, 2020). Superoxide is markedly enriched in stem cells to activate WUSCHEL (WUS) and maintain stemness, whereas H_2_O_2_ is more abundant in the differentiating peripheral zone to promote stem cell differentiation. Moreover, H_2_O_2_ negatively regulates superoxide synthesis in stem cells, and increasing H_2_O_2_ levels or scavenging superoxide leads to the termination of stem cells.

*ROS1*, which encodes one of the key DNA demethylases, was recently identified as a target for superoxide in the nucleus of stem cells. ROS1, which directly excises 5-methylcytosine from DNA, is a repressor of transcriptional gene silencing that is responsible for demethylation of the promotor of the Type-B cytokinin response regulator ARABIDOPSIS RESPONSE REGULATOR 12 (ARR12). The Fe–S clusters of ROS1 are oxidized by superoxide and so activate the DNA glycosylase/lyase activity of the enzyme, increasing ARR12 expression and contributing to meristem cell maintenance (Wang *et al.*, 2023, Preprint). ARR12 thus acts downstream of ROS1-mediated superoxide signalling to maintain stem cell fate. The *ros1* mutants had lower levels of down-regulation of mRNAs encoding two stem cell regulators, WUS and CLV3, as well as reduced SAM sizes (Wang *et al.*, 2023, Preprint).

## Conclusions and perspectives

**Fig. 2.**
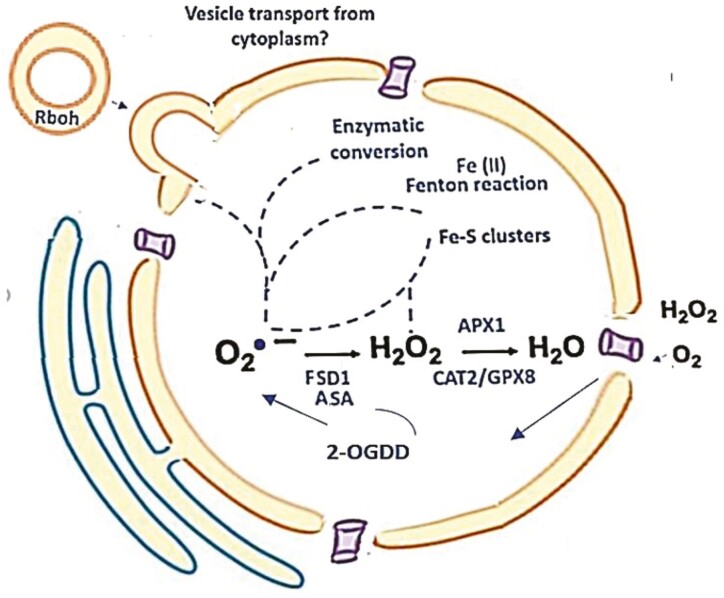
Superoxide and antioxidant processing in the nucleus. Molecular oxygen and H_2_O_2_ may enter the nucleus through the nuclear pores. The Fe–S clusters of nuclear proteins are oxidized by superoxide (O_2_·^−^). The oxidation of protein [4Fe–4S] clusters by molecular O_2_ produces Fe (II) and O_2_·^−^. Superoxide may also be generated by RBOH enzymes in the nucleus. These could be delivered to the nuclear membrane by vesicle transport from the plasma membrane. The nuclear antioxidant system includes ascorbate (AsA), GSH catalase 2 (CAT2), ascorbate peroxidase (APX1), iron superoxide dismutase 1 (FSD1), and glutathione peroxidase 8 (GPX8). 2-OGDD, Fe^2+^/2-oxoglutarate-dependent dioxygenases.

Superoxide generation by RBOH enzymes is a pivotal driver of plant growth, development, and stress responses, and yet superoxide *per se* is often ignored as a signal in favour of hydrogen peroxide, which has well characterized roles in cell signalling. Accumulating evidence suggests, however, that superoxide is generated and processed in the nucleus, as illustrated in Fig. 2. Moreover, superoxide has key regulatory and signalling functions in the nucleus, particularly in meristem maintenance and organ development, as well as other processes that incorporate multifaceted interplays between ROS and transcriptional regulators, phytohormones, and nutrients. Auxin-induced and RBOH-mediated superoxide production is important in the control of root and shoot architecture, as well as the establishment of the gravitropic curvature response in roots. Spatiotemporal regulation of the patterns of RBOH expression lead to superoxide accumulation in the apoplast in the region of the middle lamella regions of cells at pre-branch sites and LRPs during emergence, facilitating lateral root outgrowth by promoting cell wall remodelling of overlying parental tissues (Orman-Ligeza *et al.*, 2016). Superoxide participates in Fenton-type reactions in the apoplast/cell wall compartment to generate the hydroxyl radical formation required for cell wall remodelling. Moreover, the action of superoxide in the regulation of [Fe–S] cluster-containing proteins such as ROS1 is crucial to meristem maintenance and fate (Wang *et al.*, 2023, Preprint). It will be interesting to determine whether ROS1-mediated superoxide signalling is involved in the plant stress responses. The action of SODs and ascorbate in policing superoxide levels in the nucleus is thus central to superoxide signalling. However, the detection and quantitation of superoxide in the different compartments of the plant cell remain technically challenging. While there are a large number of established methods for superoxide detection, they are all problematic because of a lack of specificity. Data interpretation is therefore fraught with difficulty. The simplest are assays that measure superoxide accumulation in the apoplast/cell wall compartment of the cell, which is antioxidant poor, or when superoxide is released from cells into the surrounding solution. Most methods rely on superoxide scavengers, which react to produce a detectable product, such as the cell-permeable fluorescent probes that form the fluorescent product 2-hydroxyethidium, but none is wholly specific for superoxide (Akter *et al.*, 2021). A next generation of *in vivo* molecular probes for superoxide and hydrogen that can be targeted to specific intracellular compartments is urgently required, so that the mechanisms involved in superoxide production and metabolism in the nucleus can be fully explored.

While the movement of hydrogen peroxide across plant cell membranes through aquaporins is well documented, there is no known system for superoxide transport between different cellular compartments (Bienert and Chaumont, 2014). It is intriguing therefore that modulation of either the NADH dehydrogenase activity of respiratory chain Complex I (localized in mitochondria) or NADPH oxidase (localized in the plasma membrane) influences stem cell regulation (Zeng *et al.*, 2017). Moreover, the *atrbohD/F* double mutants had much lower levels of detectable superoxide (Zeng *et al.*, 2017). Such findings would suggest either that some of the RBOHD/F proteins are localized in the nucleus or that there are interactions between the different superoxide-generating systems in different cellular compartments. The positive relationship between nitrooleic acid, RBOH, and ROS suggests crosstalk between NO and ROS signalling (Gupta *et al.*, 2022). NO, which reacts with superoxide to form peroxynitrite (ONOO^–^) is produced in high enough concentrations to outcompete SOD for superoxide. Moreover, peroxynitrite reacts relatively slowly with most biological molecules, making peroxynitrite a suitable transport metabolite and selective oxidant. Peroxynitrite can react with CO_2_ and produce CO_3_^−^ and ·NO_2_, as well as catalysing post-translational modifications of target proteins. Taken together, these observations demonstrate that superoxide is a major driver of cell signalling that interacts with a wide range of other signalling molecules to regulate plant growth and development.

## Data Availability

This manuscript does not contain original data

## References

[CIT0001] Akter S , Khan MS, Smith EN, Flashman E. 2021. Measuring ROS and redox markers in plant cells. RSC Chemical Biology 2, 1384–1401. 34704044 10.1039/d1cb00071c PMC8495998

[CIT0002] Al-Hajaya Y , Karpinska B, Foyer CH, Baker A. 2022. Nuclear and peroxisomal targeting of catalase. Plant, Cell & Environment 45, 1096–1108. 10.1111/pce.14262 PMC9305541 35040158

[CIT0003] Andrés CMC , Pérez de la Lastra JM, Andrés Juan C, Plou FJ, Pérez-Lebeña E. 2023. Superoxide anion chemistry—its role at the core of the innate immunity. International Journal of Molecular Sciences 24, 1841. 36768162 10.3390/ijms24031841 PMC9916283

[CIT0004] Astier J , Gross I, Durner J. 2018. Nitric oxide production in plants: an update. Journal of Experimental Botany 69, 3401–3411. 29240949 10.1093/jxb/erx420

[CIT0005] Babbar R , Karpinska B, Grover A, Foyer CH. 2021. Heat-induced oxidation of the nuclei and cytosol. Frontiers in Plant Science 11, 617779. 33510759 10.3389/fpls.2020.617779 PMC7835529

[CIT0006] Bailly C. 2019. The signalling role of ROS in the regulation of seed germination and dormancy. The Biochemical Journal 476, 3019–3032. 31657442 10.1042/BCJ20190159

[CIT0007] Barragan AC , Weigel D. 2021. Plant NLR diversity: the known unknowns of pan-NLRomes. The Plant Cell 33, 814–831. 33793812 10.1093/plcell/koaa002 PMC8226294

[CIT0008] Barreto P , Dambire C, Sharma G, Vicente J, Osborne R, Yassitepe J, Gibbs DJ, Maia IG, Holdsworth MJ, Arruda P. 2022. Mitochondrial retrograde signaling through UCP1-mediated inhibition of the plant oxygen-sensing pathway. Current Biology 32, 1403–1411. 35114096 10.1016/j.cub.2022.01.037 PMC8967405

[CIT0009] Bienert GP , Chaumont F. 2014. Aquaporin-facilitated transmembrane diffusion of hydrogen peroxide. Biochimica et Biophysica Acta 1840, 1596–1604. 24060746 10.1016/j.bbagen.2013.09.017

[CIT0010] Blanco NE , Guinea-Díaz M, Whelan J, Strand Å. 2014. Interaction between plastid and mitochondrial retrograde signalling pathways during changes to plastid redox status. Philosophical Transactions of the Royal Society B: Biological Sciences 369, 20130231. 10.1098/rstb.2013.0231 PMC3949395 24591717

[CIT0011] Chapman JM , Muhlemann JK, Gayomba SR, Muday GK. 2019. RBOH-dependent ROS synthesis and ROS scavenging by plant specialized metabolites to modulate plant development and stress responses. Chemical Research in Toxicology 32, 370–396. 30781949 10.1021/acs.chemrestox.9b00028 PMC6857786

[CIT0012] Chen C , Galon Y, Ishka MR, Malihi S, Shimanovsky V, Twito S, Rath S, Vatamaniuk OK, Miller G. 2021. ASCORBATE PEROXIDASE 6 delays the onset of age-dependent leaf senescence. Plant Physiology 185, 441–456. 33580795 10.1093/plphys/kiaa031 PMC8133542

[CIT0013] Chen L , Liao B, Qi H, et al. 2015. Autophagy contributes to regulation of the hypoxia response during submergence in *Arabidopsis thaliana*. Autophagy 11, 2233–2246. 26566261 10.1080/15548627.2015.1112483 PMC4835207

[CIT0014] Considine MJ , Foyer CH. 2021. Oxygen and reactive oxygen species (ROS) dependent regulation of plant growth and development. Plant Physiology 186, 79–92. 33793863 10.1093/plphys/kiaa077 PMC8154071

[CIT0015] de Clercq I , Vermeirssen V, van Aken O, et al. 2013. The membrane-bound NAC transcription factor ANAC013 functions in mitochondrial retrograde regulation of the oxidative stress response in Arabidopsis. The Plant Cell 25, 3472–3490. 24045019 10.1105/tpc.113.117168 PMC3809544

[CIT0016] de Simone A , Hubbard R, de La Torre NV, Velappan Y, Wilson M, Considine MJ, Soppe WJJ, Foyer CH. 2017. Redox changes during the cell cycle in embryonic root meristem of *Arabidopsis thaliana*. Antioxidant & Redox Signaling 27, 1505–1519. 10.1089/ars.2016.6959 PMC5678362 28457165

[CIT0017] Diaz Vivancos P , Wolff T, Markovic J, Pallardó FV, Foyer CH. 2010. A nuclear glutathione cycle within the cell cycle. The Biochemical Journal 431, 169–178. 20874710 10.1042/BJ20100409

[CIT0018] Dietz KJ , Turkan I, Krieger-Liszkay A. 2016. Redox- and reactive oxygen species dependent signaling into and out of the photosynthesizing chloroplast. Plant Physiology 171, 1541–1550. 27255485 10.1104/pp.16.00375 PMC4936569

[CIT0019] Dolzblasz A , Nardmann J, Clerici E, Causier B, van der Graaff E, Chen J, Davies B, Werr W, Laux T. 2016. Stem cell regulation by Arabidopsis WOX genes. Molecular Plant 9, 1028–1039. 27109605 10.1016/j.molp.2016.04.007

[CIT0020] Dunand C , Crèvecoeur M, Penel C. 2007. Distribution of superoxide and hydrogen peroxide in Arabidopsis root and their influence on root development: possible interaction with peroxidases. New Phytologist 174, 332–341. 17388896 10.1111/j.1469-8137.2007.01995.x

[CIT0021] Dvořák P , Krasylenko Y, Ovečka M, Basheer J, Zapletalová V, Šamaj J, Takáč T. 2021. In vivo light-sheet microscopy resolves localisation patterns of FSD1, a superoxide dismutase with function in root development and osmoprotection. Plant, Cell & Environment 44, 68–87. 10.1111/pce.13894 32974958

[CIT0022] Eljebbawi A , del Carmen Rondón Guerrero Y, Dunand C, Estevez JM. 2020. Highlighting reactive oxygen species (ROS) as multitaskers in root development. iScience 24, 101978. 33490891 10.1016/j.isci.2020.101978 PMC7808913

[CIT0023] Emmert S , Quargnali G, Thallmai S, Rivera-Fuentes P. 2023. A locally activatable sensor for robust quantification of organellar glutathione. Nature Chemistry 15, 1415–1421. 10.1038/s41557-023-01249-3 PMC10533397 37322101

[CIT0024] Eysholdt-Derzsó E , Sauter M, Eysholdt-Derzso E, Sauter M. 2017. Root bending is antagonistically affected by hypoxia and ERF-mediated transcription via auxin signaling. Plant Physiology 175, 412–423. 28698356 10.1104/pp.17.00555 PMC5580755

[CIT0025] Fichman Y , Rowland L, Oliver MJ, Mittler R. 2023. ROS are evolutionary conserved cell-to-cell stress signals. Proceedings of the National Academy of Sciences, USA 120, e2305496120. 10.1073/pnas.2305496120 PMC10400990 37494396

[CIT0026] Fichman Y , Zandalinas SI, Peck S, Luan S, Mittler R. 2022. HPCA1 is required for systemic reactive oxygen species and calcium cell-to-cell signaling and plant acclimation to stress. The Plant Cell 34, 4453–4471. 35929088 10.1093/plcell/koac241 PMC9724777

[CIT0027] Foyer CH , Baker A, Wright M, Sparkes I, Mhamdi A, Schippers JHM, Van Breusegem F. 2020. On the move: redox-dependent protein relocation. Journal of Experimental Botany 71, 620–631. 31421053 10.1093/jxb/erz330

[CIT0028] Foyer CH , Hanke G. 2022. ROS production and signalling in chloroplasts: cornerstones and evolving concepts. The Plant Journal 111, 642–661. 35665548 10.1111/tpj.15856 PMC9545066

[CIT0029] Fuentes-Lemus E , Davies MJ. 2023. Effect of crowding, compartmentalization and nanodomains on protein modification and redox signaling—current state and future challenges. Free Radical Biology and Medicine 196, 81–92. 36657730 10.1016/j.freeradbiomed.2023.01.011

[CIT0030] Gaber A , Ogata T, Maruta T, Yoshimura K, Tamoi M, Shigeoka S. 2012. The involvement of Arabidopsis glutathione peroxidase 8 in the suppression of oxidative damage in the nucleus and cytosol. Plant and Cell Physiology 53, 1596–1606. 22773682 10.1093/pcp/pcs100

[CIT0031] García-Giménez JL , Markovic J, Dasí F, Queval G, Schnaubelt D, Foyer CH, Pallardó FV. 2013. Nuclear glutathione. Biochimica et Biophysica Acta 1830, 3304–3316. 23069719 10.1016/j.bbagen.2012.10.005

[CIT0032] Giraud E , van Aken O, Ho LHM, Whelan J. 2009. The transcription factor ABI4 is a regulator of mitochondrial retrograde expression of ALTERNATIVE OXIDASE1a. Plant Physiology 150, 1286–1296. 19482916 10.1104/pp.109.139782 PMC2705018

[CIT0033] Gonzali S , Loreti E, Cardarelli F, Novi G, Parlanti S, Pucciariello C, Bassolino L, Banti V, Licausi F, Perata P. 2015. Universal stress protein HRU1 mediates ROS homeostasis under anoxia. Nature Plants 1, 15151. 27251529 10.1038/nplants.2015.151

[CIT0034] Gray B , Carmichael AJ. 1992. Kinetics of superoxide scavenging by dismutase enzymes and manganese mimics determined by electron spin resonance. The Biochemical Journal 281, 795–802. 1311175 10.1042/bj2810795 PMC1130760

[CIT0035] Großkinsky DK , Koffler BE, Roitsch T, Maier R, Zechmann B. 2012. Compartment specific antioxidative defense in Arabidopsis against virulent and avirulent *Pseudomonas syringae*. Phytopathology 1, 662–673. 10.1094/PHYTO-02-12-0022-R PMC3822284 22571419

[CIT0036] Gupta KJ , Kaladhar VC, Fitzpatrick TB, Fernie AR, Moller IM, Loake GJ. 2022. Nitric oxide regulation of plant metabolism. Molecular Plant 15, 228–242. 34971792 10.1016/j.molp.2021.12.012

[CIT0037] Gutsche N , Holtmannspötter M, Maß L, O’Donoghue M, Busch A, Lauri A, Schubert V, Zachgo S. 2017. Conserved redox-dependent DNA binding of ROXY glutaredoxins with TGA transcription factors. Plant Direct 1, e00030. 31245678 10.1002/pld3.30 PMC6508501

[CIT0038] Harris W , Kim S, Völz R, Lee YH. 2023. Nuclear effectors of plant pathogens: distinct strategies to be one step ahead. Molecular Plant Pathology 24, 637–650. 36942744 10.1111/mpp.13315 PMC10189769

[CIT0039] He H , Van Breusegem F, Mhamdi A. 2018. Redox-dependent control of nuclear transcription in plants. Journal of Experimental Botany 69, 3359–3372. 29659979 10.1093/jxb/ery130

[CIT0040] Huang F , Luo X, Ou Y, Gao Z, Tang Q, Chu Z, Zhu X, He Y. 2023. Control of histone demethylation by nuclear-localized α-ketoglutarate dehydrogenase. Science 381, 8822. 10.1126/science.adf8822 37440635

[CIT0041] Huang S , Van Aken O, Schwarzländer M, Belt K, Millar AH. 2016. The roles of mitochondrial reactive oxygen species in cellular signaling and stress response in plants. Plant Physiology 171, 1551–1559. 27021189 10.1104/pp.16.00166 PMC4936549

[CIT0042] Islam BU , Habib S, Ahmad P, Allarakha S, Moinuddin, Ali A. 2015. Pathophysiological role of peroxynitrite induced DNA damage in human diseases: a special focus on poly(ADP-ribose) polymerase (PARP). Indian Journal of Clinical Biochemistry 30, 368–385. 26788021 10.1007/s12291-014-0475-8 PMC4712174

[CIT0043] Jiang K , Zhu T, Diao Z, Huang H, Feldman LJ. 2010. The maize root stem cell niche: a partnership between two sister cell populations. Planta 231, 411–424. 20041334 10.1007/s00425-009-1059-3 PMC2799627

[CIT0044] Jones JD , Dangl JL. 2006. The plant immune system. Nature 444, 323–329. 17108957 10.1038/nature05286

[CIT0045] Jurdak R , Launay-Avon A, Paysant-le R, Bailly C. 2021. Retrograde signalling from the mitochondria to the nucleus translates the positive effect of ethylene on dormancy breaking of *Arabidopsis thaliana* seeds. New Phytologist 229, 2192–2205. 33020928 10.1111/nph.16985

[CIT0046] Karpinska B , Owdah Alomrani S, Foyer CH. 2017. Inhibitor-induced oxidation of the nucleus and cytosol in *Arabidopsis thaliana*: implications for organelle to nucleus retrograde signalling. Philosophical Transactions of the Royal Society B: Biological Sciences 372, 20160392. 10.1098/rstb.2016.0392 PMC5566886 28808105

[CIT0047] Kim JB , Sebastiano V, Wu G, et al. 2009. Oct4-induced pluripotency in adult neural stem cells. Cell 136, 411–419. 19203577 10.1016/j.cell.2009.01.023

[CIT0048] Kneeshaw S , Keyani R, Delorme-Hinoux V, Imrie L, Loake GJ, Le Bihan T, Reichheld JP, Spoel SH. 2017. Nucleoredoxin guards against oxidative stress by protecting antioxidant enzymes. Proceedings of the National Academy of Sciences, USA 114, 8414–8419. 10.1073/pnas.1703344114 PMC5547615 28724723

[CIT0049] Koffler BE , Luschin-Ebengreuth N, Stabentheiner E, Müller M, Zechmann B. 2014a. Compartment specific response of antioxidants to drought stress in Arabidopsis. Plant Science 227, 133–144. 25219315 10.1016/j.plantsci.2014.08.002 PMC4180016

[CIT0050] Koffler BE , Luschin-Ebengreuth N, Zechmann B. 2015. Compartment specific changes of the antioxidative status in *Arabidopsis thaliana* during salt stress. Journal of Plant Biology 58, 8–16.

[CIT0051] Koffler BE , Polanschütz LM, Zechmann B. 2014b. Higher sensitivity of pad2-1 and vtc2-1 mutants to cadmium is related to lower subcellular glutathione rather than ascorbate contents. Protoplasma 251, 755–769. 24281833 10.1007/s00709-013-0576-x PMC4059996

[CIT0052] Kosmacz M , Parlanti S, Schwarzländer M, Kragler F, Licausi F, Van Dongen JT. 2015. The stability and nuclear localization of the transcription factor RAP2.12 are dynamically regulated by oxygen concentration. Plant, Cell & Environment 38, 1094–1103. 10.1111/pce.12493 25438831

[CIT0053] Kuroda J , Nakagawa K, Yamasaki T, et al. 2005. The superoxide-producing NAD(P)H oxidase Nox4 in the nucleus of human vascular endothelial cells. Genes To Cells 10, 1139–1151. 16324151 10.1111/j.1365-2443.2005.00907.x

[CIT0054] Lee J , Han M, Shin Y, Lee J, Heo G, Lee Y. 2023. How extracellular reactive oxygen species reach their intracellular targets in plants. Molecules and Cells 46, 329–336. 36799103 10.14348/molcells.2023.2158 PMC10258463

[CIT0055] Li G , Liu J, Guan Y, Ji X. 2021. The role of hypoxia in stem cell regulation of the central nervous system: from embryonic development to adult proliferation. CNS Neuroscience & Therapeutics 27, 1446–1457. 34817133 10.1111/cns.13754 PMC8611781

[CIT0056] Li S , Lauri A, Ziemann M, Busch A, Bhave M, Zachgo S. 2009. Nuclear activity of ROXY1, a glutaredoxin interacting with TGA factors, is required for petal development in *Arabidopsis thaliana*. The Plant Cell 21, 429–441. 19218396 10.1105/tpc.108.064477 PMC2660636

[CIT0057] Liu B , Sun L, Ma L, Hao F-SS. 2017. Both AtRBOHD and AtRBOHF are essential for mediating responses to oxygen deficiency in Arabidopsis. Plant Cell Reports 36, 1–11. 28337518 10.1007/s00299-017-2128-x

[CIT0058] Liu J-X , Feng, K Duan A-Q, Li H, Yang Q-Q, Xu Z-S, Xiong A-S. 2019. Isolation, purification and characterization of an ascorbate peroxidase from celery and overexpression of the AgAPX1 gene enhanced ascorbate content and drought tolerance in Arabidopsis. BMC Plant Biology 19, 488–499. 31711410 10.1186/s12870-019-2095-1 PMC6849298

[CIT0059] Lyublinskaya OG , Borisov YG, Pugovkina NA, et al. 2015. Reactive oxygen species are required for human mesenchymal stem cells to initiate proliferation after the quiescence exit. Oxidative Medicine and Cellular Longevity 2015, 502105. 26273423 10.1155/2015/502105 PMC4530296

[CIT0060] Mahmood AM , Dunwell JM. 2019. Evidence for novel epigenetic marks within plants. AIMS Genetics 6, 70–87. 31922011 10.3934/genet.2019.4.70 PMC6949463

[CIT0061] Mailloux RJ. 2020. An update on mitochondrial reactive oxygen species production. Antioxidants (Basel) 9, 472. 32498250 10.3390/antiox9060472 PMC7346187

[CIT0062] Martins L , Trujillo-Herandez JA, Reichheld J-P. 2018. Thiol based redox signalling in the plant nucleus. Frontiers in Plant Science 9, 705. 29892308 10.3389/fpls.2018.00705 PMC5985474

[CIT0063] Marzec-Schmidt K , Wojciechowska N, Nemeczek K, Ludwików A, Mucha J, Bagniewska-Zadworna A. 2020. Allies or enemies: the role of reactive oxygen species in developmental processes of black cottonwood (*Populus trichocarpa*). Antioxidants 9, 199. 32120843 10.3390/antiox9030199 PMC7139288

[CIT0064] Maß L , Holtmannspötter M, Zachgo S. 2020. Dual-color 3D–dSTORM colocalization and quantification of ROXY1 and RNAPII variants throughout the transcription cycle in root meristem nuclei. The Plant Journal 104, 1423–1436. 32896918 10.1111/tpj.14986

[CIT0065] Melicher P , Dvořák P, Krasylenko Y, Shapiguzov A, Kangasjärvi J, Šamaj J, Takáč T. 2022. Arabidopsis iron superoxide dismutase FSD1 protects against methyl viologen-induced oxidative stress in a copper-dependent manner. Frontiers in Plant Science 13, 823561. 35360337 10.3389/fpls.2022.823561 PMC8963501

[CIT0066] Michelet L , Krieger-Liszkay A. 2012. Reactive oxygen intermediates produced by photosynthetic electron transport are enhanced in short-day grown plants. Biochimica Biophysica Acta 1817, 1306–1313. 10.1016/j.bbabio.2011.11.014 22172734

[CIT0067] Miller G , Mittler R. 2023. Plant NADPH oxidases. In: Pick E, ed. NADPH oxidases revisited: from function to structure. Cham: Springer, 445–465.

[CIT0068] Mira MM , El-Khateeb EA, Gaafar RM, Igamberdiev AB, Hill RD, Stasolla C. 2020. Stem cell fate in hypoxic root apical meristems is influenced by phytoglobin expression. Journal of Experimental Botany 71, 1350–1362. 31541257 10.1093/jxb/erz410

[CIT0069] Mittler R , Zandalinas SI, Fichman Y, Van Breusegem F. 2022. Reactive oxygen species signalling in plant stress responses. Nature Reviews. Molecular Cell Biology 23, 663–679. 35760900 10.1038/s41580-022-00499-2

[CIT0070] Moller IM. 2001. Plant mitochondria and oxidative stress: electron transport, NADPH turnover, and metabolism of reactive oxygen species. Annual Review of Plant Physiology and Plant Molecular Biology 52, 561–591. 10.1146/annurev.arplant.52.1.561 11337409

[CIT0071] Orman-Ligeza B , Parizot B, de Rycke R, Fernandez A, Himschoot E, Van Breusegem F, Bennett MJ, Périlleux C, Beeckman T, Draye X. 2016. RBOH-mediated ROS production facilitates lateral root emergence in Arabidopsis. Development 143, 3328–3339. 27402709 10.1242/dev.136465 PMC5047660

[CIT0072] Paszkiewicz G , Gualberto JM, Benamar A, Macherel D, Logan DC. 2017. Arabidopsis seed mitochondria are bioenergetically active immediately upon imbibition and specialize via biogenesis in preparation for autotrophic growth. The Plant Cell 29, 109–128. 28062752 10.1105/tpc.16.00700 PMC5304351

[CIT0073] Pucciariello C , Perata P. 2021. The oxidative paradox in low oxygen stress in plants. Antioxidants (Basel) 10, 332. 33672303 10.3390/antiox10020332 PMC7926446

[CIT0074] Ratti M , Lampis A, Ghidini M, Salati M, Mirchev MB, Valeri N, Hahne JC. 2020. MicroRNAs (miRNAs) and long non-coding RNAs (lncRNAs) as new tools for cancer therapy: first steps from bench to bedside. Targeted Oncology 15, 261–278. 32451752 10.1007/s11523-020-00717-x PMC7283209

[CIT0075] Richards SL , Wilkins KA, Swarbreck SM, Anderson AA, Habib N, Smith AG, McAinsh M, Davies, JM. 2015. The hydroxyl radical in plants: from seed to seed. Journal of Experimental Botany 66, 37–46. 25294918 10.1093/jxb/eru398

[CIT0076] Sandalio LM , Peláez-Vico MA, Molina-Moya E, Romero-Puertas MC. 2021. Peroxisomes as redox-signaling nodes in intracellular communication and stress responses. Plant Physiology 186, 22–35. 33587125 10.1093/plphys/kiab060 PMC8154099

[CIT0077] Sankaranarayanan S , Ju Y, Kessler SA. 2020. Reactive oxygen species as mediators of gametophyte development and double fertilization in flowering plants. Frontiers in Plant Science 11, 1199. 32849744 10.3389/fpls.2020.01199 PMC7419745

[CIT0078] Sasidharan R , Schippers JHM, Schmidt RR. 2021. Redox and low-oxygen stress: signal integration and interplay. Plant Physiology 186, 66–78. 33793937 10.1093/plphys/kiaa081 PMC8154046

[CIT0079] Scandroglio F , Tórtora V, Radi R, Castro L. 2014. Metabolic control analysis of mitochondrial aconitase: influence over respiration and mitochondrial superoxide and hydrogen peroxide production. Free Radical Research 48, 684–693. 24601712 10.3109/10715762.2014.900175

[CIT0080] Schippers JH , Foyer CH, van Dongen JT. 2016. Redox regulation in shoot growth, SAM maintenance and flowering. Current Opinion in Plant Biology 29, 121–128. 26799134 10.1016/j.pbi.2015.11.009

[CIT0081] Schmidt R , Schippers JH. 2015. ROS-mediated redox signaling during cell differentiation in plants. Biochimica et Biophysica Acta 1850, 1497–1508. 25542301 10.1016/j.bbagen.2014.12.020

[CIT0082] Shapiguzov A , Vainonen JP, Hunter K, et al. 2019. *Arabidopsis* RCD1 coordinates chloroplast and mitochondrial functions through interaction with ANAC transcription factors. eLife 8, e43284. 30767893 10.7554/eLife.43284 PMC6414205

[CIT0083] Sheng Y , Abreu IA, Cabelli DE, Maroney MJ, Miller AF, Teixeira M, Valentine JS. 2014. Superoxide dismutases and superoxide reductases. Chemical Reviews 114, 3854–3918. 24684599 10.1021/cr4005296 PMC4317059

[CIT0084] Shukla V , Lombardi L, Iacopino S, Pencik A, Novak O, Perata P, Giuntoli B, Licausi F. 2019. Endogenous hypoxia in lateral root primordia controls root architecture by antagonizing auxin signaling in Arabidopsis. Molecular Plant 12, 538–551. 30641154 10.1016/j.molp.2019.01.007

[CIT0085] Simon UK , Polanschütz LM, Koffler BE, Zechmann B. 2013. High resolution imaging of temporal and spatial changes of subcellular ascorbate, glutathione and H_2_O_2_ distribution during *Botrytis cinerea* infection in Arabidopsis. PLoS One 8, e65811. 23755284 10.1371/journal.pone.0065811 PMC3673919

[CIT0086] Soldner F , Hockemeyer D, Beard C, et al. 2009. Parkinson’s disease patient-derived induced pluripotent stem cells free of viral reprogramming factors. Cell 136, 964–977. 19269371 10.1016/j.cell.2009.02.013 PMC2787236

[CIT0087] Som S , Raha C, Chatterjee IB. 1983. Ascorbic acid: a scavenger of superoxide radical. Acta Vitaminologica et Enzymologica 5, 243–250. 6324567

[CIT0088] Szechyńska-Hebda M , Lewandowska M, Witoń D, Fichman Y, Mittler R, Karpiński SM. 2023. Aboveground plant-to-plant electrical signaling mediates network acquired acclimation. The Plant Cell 34, 3047–3065. 10.1093/plcell/koac150 PMC9338792 35595231

[CIT0089] Tanaka K , Heil M. 2021. Damage-associated molecular patterns (DAMPs) in plant innate immunity: applying the danger model and evolutionary perspectives. Annual Review of Phytopathology 59, 53–75. 10.1146/annurev-phyto-082718-100146 33900789

[CIT0090] Tao J , Wu F, Wen H, et al. 2023. RCD1 promotes salt stress tolerance in *Arabidopsis* by repressing ANAC017 activity. International Journal of Molecular Sciences 24, 9793. 37372941 10.3390/ijms24129793 PMC10298584

[CIT0091] Tsang CK , Liu Y, Thomas J, Zhang Y, Zheng XF. 2014. Superoxide dismutase 1 acts as a nuclear transcription factor to regulate oxidative stress resistance. Nature Communications 19, 3446. 10.1038/ncomms4446 PMC4678626 24647101

[CIT0092] Tsukagoshi H , Busch W, Benfey PN. 2010. Transcriptional regulation of ROS controls transition from proliferation to differentiation in the root. Cell 143, 606–616. 21074051 10.1016/j.cell.2010.10.020

[CIT0093] Vanderauwera S , Vandenbroucke K, Inzé A, van de Cotte B, Mühlenbock P, De Rycke R, Naouar N, Van Gaever T, Van Montagu MCE, Van Breusegem F. 2012. AtWRKY15 perturbation abolishes the mitochondrial stress response that steers osmotic stress tolerance in Arabidopsis. Proceedings of the National Academy of Sciences, USA 109, 20113–20118. 10.1073/pnas.1217516109 PMC3523852 23169634

[CIT0094] Vitor SC , Duarte GT, Saviani EE, Vincentz MG, Oliveira HC, Salgado I. 2013. Nitrate reductase is required for the transcriptional modulation and bactericidal activity of nitric oxide during the defense response of *Arabidopsis thaliana* against *Pseudomonas syringae*. Planta 238, 475–486. 23748675 10.1007/s00425-013-1906-0

[CIT0095] Wagner S , van Aken O, Elsässer M, Schwarzländer M. 2018. Mitochondrial energy signaling and its role in the low-oxygen stress response of plants. Plant Physiology 176, 1156–1170. 29298823 10.1104/pp.17.01387 PMC5813528

[CIT0096] Wang C , El-Shetehy M, Shine MB, Yu K, Navarre D, Wendehenne D, Kachroo A, Kachroo P. 2014. Free radicals mediate systemic acquired resistance. Cell Reports 7, 348–355. 24726369 10.1016/j.celrep.2014.03.032

[CIT0097] Wang S , Liu M, Hu D, Dong Z, Zhao Z 2023. Control of DNA demethylation by superoxide anion in plant stem cells. Research Square 10.21203/rs.3.rs-3313783/v1 [Preprint]. 39266722

[CIT0098] Wang Y , Selinski J, Mao C, Zhu Y, Berkowitz O, Whelan J. 2020. Linking mitochondrial and chloroplast retrograde signalling in plants. Philosophical Transactions of the Royal Society B: Biological Sciences 375, 20190410. 10.1098/rstb.2019.0410 PMC7209950 32362265

[CIT0099] Wang Z , Sun J, Zu X, et al. 2022. Pseudouridylation of chloroplast ribosomal RNA contributes to low temperature acclimation in rice. New Phytologist 236, 1708–1720. 36093745 10.1111/nph.18479

[CIT0100] Wany A , Gupta KJ. 2018. Reactive oxygen species, nitric oxide production and antioxidant gene expression during development of aerenchyma formation in wheat. Plant Signaling and Behavior 13, e1428515. 29336716 10.1080/15592324.2018.1428515 PMC5846502

[CIT0101] Waszczak C , Carmody M, Kangasjärvi J. 2018. Reactive oxygen species in plant signaling. Annual Review of Plant Biology 69, 209–236. 10.1146/annurev-arplant-042817-040322 29489394

[CIT0102] Wei S , Zhang W, Fu R, Zhang Y. 2021. Genome-wide characterization of 2-oxoglutarate and Fe(II)-dependent dioxygenase family genes in tomato during growth cycle and their roles in metabolism. BMC Genomics 22, 126. 33602133 10.1186/s12864-021-07434-3 PMC7891033

[CIT0103] Weits DA , Kunkowska AB, Kamps NCW, et al. 2023. An apical hypoxic niche sets the pace of shoot meristem activity. Nature 569, 716–718. 10.1038/s41586-019-1203-6 31092919

[CIT0104] Wing CE , Fung HYJ, Chook YM. 2022. Karyopherin-mediated nucleocytoplasmic transport. Nature Reviews. Molecular Cell Biology 23, 307–328. 35058649 10.1038/s41580-021-00446-7 PMC10101760

[CIT0105] Wirthmueller L , Asai S, Rallapalli G, et al. 2018. Arabidopsis downy mildew effector HaRxL106 suppresses plant immunity by binding to RADICAL-INDUCED CELL DEATH1. New Phytologist 220, 232–248. 30156022 10.1111/nph.15277 PMC6175486

[CIT0106] Wurm CJ , Lindermayr C. 2021. Nitric oxide signaling in the plant nucleus: the function of nitric oxide in chromatin modulation and transcription. Journal of Experimental Botany 72, 808–818. 33128375 10.1093/jxb/eraa404

[CIT0107] Xu J , Su X, Burley SK, Zheng XFS. 2022. Nuclear SOD1 in growth control, oxidative stress response, amyotrophic lateral sclerosis, and cancer. Antioxidants 11, 427. 35204309 10.3390/antiox11020427 PMC8869091

[CIT0108] Yang W , Hekimi S. 2010. A mitochondrial superoxide signal triggers increased longevity in *Caenorhabditis elegans*. PLoS Biology 8, e1000556. 21151885 10.1371/journal.pbio.1000556 PMC2998438

[CIT0109] Yeung E , van Veen H, Vashisht D, et al, 2018. A stress recovery signaling network for enhanced flooding tolerance in *Arabidopsis thaliana*. Proceedings of the National Acadeny of Sciences USA 115, E6085–E6094. 10.1073/pnas.1803841115 PMC6042063 29891679

[CIT0110] Zachgo S. 2023. Nuclear redox processes in land plant development and stress adaptation. Biological Chemistry 404, 379–384. 36853884 10.1515/hsz-2022-0288

[CIT0111] Zechmann B. 2011. Subcellular distribution of ascorbate in plants. Plant Signaling & Behavior 6, 360–363. 21350341 10.4161/psb.6.3.14342 PMC3142415

[CIT0112] Zechmann B. 2014. Compartment-specific importance of glutathione during abiotic and biotic stress. Frontiers in Plant Science 5, 566. 25368627 10.3389/fpls.2014.00566 PMC4202713

[CIT0113] Zechmann B. 2018. Compartment-specific importance of ascorbate during environmental stress in plants. Antioxidants and Redox Signalling 29, 1488–1501. 10.1089/ars.2017.7232 28699398

[CIT0114] Zechmann B. 2020. Subcellular roles of glutathione in mediating plant defense during biotic stress. Plants 9, 1067. 32825274 10.3390/plants9091067 PMC7569779

[CIT0115] Zechmann B , Stumpe M, Mauch F. 2011. Immunocytochemical determination of the subcellular distribution of ascorbate in plants. Planta 233, 1–12. 20872269 10.1007/s00425-010-1275-x PMC3015205

[CIT0116] Zeng J , Dong Z, Wu H, Tian Z, Zhao Z. 2017. Redox regulation of plant stem cell fate. The EMBO Journal 36, 2844–2855. 28838936 10.15252/embj.201695955 PMC5623875

[CIT0117] Zhang M , Li Q, Liu T, Liu L, Shen D, Zhu Y, Liu P, Zhou JM, Dou D. 2015. Two cytoplasmic effectors of *Phytophthora sojae* regulate plant cell death via interactions with plant catalases. Plant Physiology 167, 164–175. 25424308 10.1104/pp.114.252437 PMC4281015

[CIT0118] Zhang W , Chen SJ, Guo LY, et al. 2023. Nitric oxide synthase and its function in animal reproduction: an update. Frontiers in Physiology 14, 1288669. 38028794 10.3389/fphys.2023.1288669 PMC10662090

[CIT0119] Zhithovich A. 2020. Nuclear and cytoplasmic functions of vitamin C. Chemical Research in Toxicology 33, 2515–2526. 33001635 10.1021/acs.chemrestox.0c00348 PMC7572711

[CIT0120] Zhou X , Xiang Y, Li C, Yu G. 2020. Modulatory role of reactive oxygen species in root development in model plant of *Arabidopsis thaliana*. Frontiers in Plant Science 11, 485932. 33042167 10.3389/fpls.2020.485932 PMC7525048

[CIT0121] Zur I , Kopeć P, Surówka E, et al. 2021. Impact of ascorbate–glutathione cycle components on the effectiveness of embryogenesis induction in isolated microspore cultures of barley and triticale. Antioxidants 10, 1254. 34439502 10.3390/antiox10081254 PMC8389252

